# *Lacticaseibacillus rhamnosus* alleviates intestinal inflammation and promotes microbiota-mediated protection against *Salmonella* fatal infections

**DOI:** 10.3389/fimmu.2022.973224

**Published:** 2022-08-11

**Authors:** Xianqi Peng, Abdelaziz Ed-Dra, Yan Song, Mohammed Elbediwi, Reshma B. Nambiar, Xiao Zhou, Min Yue

**Affiliations:** ^1^ Department of Veterinary Medicine and Institute of Preventive Veterinary Sciences, College of Animal Science, Zhejiang University, Hangzhou, China; ^2^ Hainan Institute of Zhejiang University, Sanya, China; ^3^ Zhejiang Provincial Key Laboratory of Preventive Veterinary Medicine, Hangzhou, China; ^4^ State Key Laboratory for Diagnosis and Treatment of Infectious Diseases, National Clinical Research Center for Infectious Diseases, National Medical Center for Infectious Diseases, The First Affiliated Hospital, College of Medicine, Zhejiang University, Hangzhou, China

**Keywords:** *Lacticaseibacillus rhamnosus*, intestinal inflammation, microbiota, *Salmonella typhimurium*, infection

## Abstract

The fatal impairment of the intestinal mucosal barrier of chicks caused by *Salmonella* significantly resulting economic losses in the modern poultry industry. Probiotics are recognized for beneficially influencing host immune responses, promoting maintenance of intestinal epithelial integrity, antagonistic activity against pathogenic microorganisms and health-promoting properties. Some basic studies attest to probiotic capabilities and show that *Lacticaseibacillus rhamnosus* could protect intestinal mucosa from injury in animals infected with *Salmonella* Typhimurium. However, the mechanisms underlying its protective effects in chicks are still not fully understood. Here, we used the chick infection model combined with histological, immunological, and molecular approaches to address this question. The results indicated that *L. rhamnosus* significantly reduced the diarrhea rate and increased the daily weight gain and survival rate of chicks infected with *S.* Typhimurium. Furthermore, we found that *L. rhamnosus* markedly improved the immunity of gut mucosa by reducing apoptotic cells, hence effectively inhibiting intestinal inflammation. Notably, pre-treatment chicks with *L. rhamnosus* balanced the expression of interleukin-1β and interleukin-18, moderated endotoxin and D-lactic acid levels, and expanded tight junction protein levels (Zonula occluden-1 and Claudin-1), enhanced the function of the intestinal mucosal epithelial cells. Additionally, investigations using full-length 16S rRNA sequencing also demonstrated that *L. rhamnosus* greatly weakened the adhesion of *Salmonella*, the mainly manifestation is the improvement of the diversity of intestinal microbiota in infected chicks. Collectively, these results showed the application of *L. rhamnosus* against *Salmonella* fatal infection by enhancing barrier integrity and the stability of the gut microbiota and reducing inflammation in new hatch chicks, offering new antibiotic alternatives for farming animals.

## Introduction

*Salmonella* is a Gram-negative bacterium belonging to the Enterobacteriaceae family. It has been recognized as one of the most common pathogens causing gastroenteritis and systemic infections to chickens (1), resulting in a substantial economic loss to poultry industries (2–4). To date, at least 2,659 serovars have been described (5), where *S.* Gallinarum biovars Gallinarum and Pullorum have been considered the usual agents causing infections in chickens (4, 6–9). However, the implementation of successful programs has tackled the emergence of *S.* Gallinarum in poultry farms, especially in developed and some developing countries (10, 11). Nevertheless, recent surveillance studies have reported the involvement of non-host specific *Salmonella* serovars in many chicken infections, including serovars Typhimurium, Enteritidis, Thompson, and others (2, 12–14), which challenge efforts provided to ensure the safety of chicken herds.

*Salmonella* infections occur *via* fecal-oral transmission, where the pathogen overcomes multilayered intestinal barriers (from the intestinal lumen to the tissue: digestive juice, antibacterial substances secreted by symbiotic bacteria, water layer, glycocalyx, and mucus layer, etc.) to interact with the intestinal epithelium and penetrate deeper into the tissues of the host (13, 15–20). The intestinal epithelial barrier (IEB) is the body’s first line of defense against the invasion of harmful substances and pathogens in the external environment, composed of a mucus layer, a single cell layer of epithelial cells and an inherent layer rich in immune cells (15). A series of different cellular junctions, including tight junctions (TJs), adherence junctions (AJs), gap junctions and desmosomes, are essential to the proper functioning of the IEB (21). The AJC is a crucial node maintaining epithelial permeability by selectively limiting the paracellular diffusion (15, 22, 23). Previous studies have shown that *Salmonella* adheres to epithelial tissue and destroys cell junctions, leading to intestinal inflammation and diarrhea (24–26). Hence, maintaining the integrity of epithelial tissue may reduce the pathogenicity and severity of *Salmonella* infections.

There is an interaction between the IEB homeostasis and the intestinal microbiota; the intestinal permeability is affected by regulating the expression and assembly of TJs between intestinal flora and epithelial cells. Birds’ gut microbiota is changing from a migrant community to one that becomes increasingly complex as they grow. Proteobacteria is the predominant phylum in the new hatch chicks. With the growth, the dominant position is gradually replaced by Firmicutes and Tenericutes (>14 days). The structure of the microbial community of the older birds have become significantly more diverse and the intestinal homeostasis gradually formed (27).

Probiotics are live microorganisms that, when administered adequately, confer health benefits on the host (28). In fact, one of the essential cytoprotective effects of probiotics in the intestinal mucosa is enhancing epithelial barrier functions through the regulation of cellular junctions (29, 30). In this regard, Yu et al. demonstrated the ability of *Fructobacillus fructosus* C2 to protect the integrity of Caco-2 cells against the damage caused by enterotoxigenic *E. coli* (ETEC) and *S.* Typhimurium infections (31). Additionally, Hummel et al. found that some lactic acid bacteria like *Lactobacillus acidophilus, Lactobacillus gasseri, Limosilactobacillus fermentum*, and *Lacticaseibacillus rhamnosus*, these agents increased transepithelial resistance in T84 cells and modulated the expression of E-cadherin and β-catenin (adherence junction proteins) (23). Moreover, the probiotic mixture BEC showed the ability to increase the laying rate and prevent the decrease in expression and redistribution of the tight junction proteins occludin, zonula occluden-1 (ZO-1) and Mucin-2 in coccidia and *Clostridium perfringens* challenge in laying hens (32). Mennigen et al. (33) found that a murine model of colitis was able to decrease apoptosis by administering the probiotic mixture VSL#3, which is another important pathway to enhance epithelial barrier functions. Recently, Yang et al. (34) have demonstrated the efficacity of *Bifidobacterium lactis* JYBR-190 in protecting intestinal mucosal damage caused by *Salmonella* Pullorum in chicks. Importantly, it has been shown that some probiotics may participate in promoting immune response in chicks, where Hu et al. have proved the efficacity of *Limosilactobacillus reuteri* in suppressing the LPS-induced expression of pro-inflammatory genes (IL-1β and IL-8), and increasing the expression of anti-inflammatory genes (TGF-β and IL-10) in the duodenum in broilers (35).

*L. rhamnosus* is recognized as a safe probiotic and has been used in different fields for its benefits on both human and animal health (36). According to some newly published articles, *L. rhamnosus* MTCC-5897 possessed protective effects on intestinal epithelial barrier function in a colitis-induced murine model (37). Additionally, *L. rhamnosus* FBB81 enhanced intestinal epithelial barrier function in an *in vitro* model of hydrogen peroxide-induced inflammatory bowel disease (38). However, to our knowledge, the beneficial effect of *L. rhamnosus* on the intestinal barrier function of newly hatched chicks challenged with *Salmonella* Typhimurium has not yet been studied. Therefore, we aim in this study to evaluate the ability of *L. rhamnosus* P118 to restore the intestinal barrier disrupted by *Salmonella* Typhimurium infection in chicks, in which we will investigate the effect of probiotic *L. rhamnosus* P118 on the gut’s internal environment stability in the presence and absence of *Salmonella* infection.

## Materials and methods

### Bacterial isolates, culture media, and growth conditions

The potential probiotic strain used in this study was isolated and confirmed previously in our lab. Briefly, strains of lactic acid bacteria (LAB) were isolated from fermented yoghurt, confirmed by whole genome sequencing (WGS) and compared with the GenBank. A total of 292 LAB strains were isolated, the *L. rhamnosus* P118 strain (PRJNA848987) for this study was selected based on preliminary results obtained *in vitro* and *in silico*. To obtain the inoculum of the LAB, *L. rhamnosus* strain P118 was propagated twice in the DeMan, Rogosa, and Sharpe (MRS, Oxoid Ltd, Hants, UK) broth at 37°C for 16h under shaking. The concentration of bacterial suspension was measured by spectrophotometer and presented as equivalent to CFU/mL. Moreover, a spontaneous novobiocin-resistant *S.* Typhimurium SL1344 kept in our lab was grown overnight in the Luria-Bertani (LB) broth at 37 °C in an orbital shaking incubator at 180 rpm/min, sub-cultured twice, and then the CFU/mL was determined by spectrophotometer.

### Chicks’ management and experimental design

**Figure 1:**
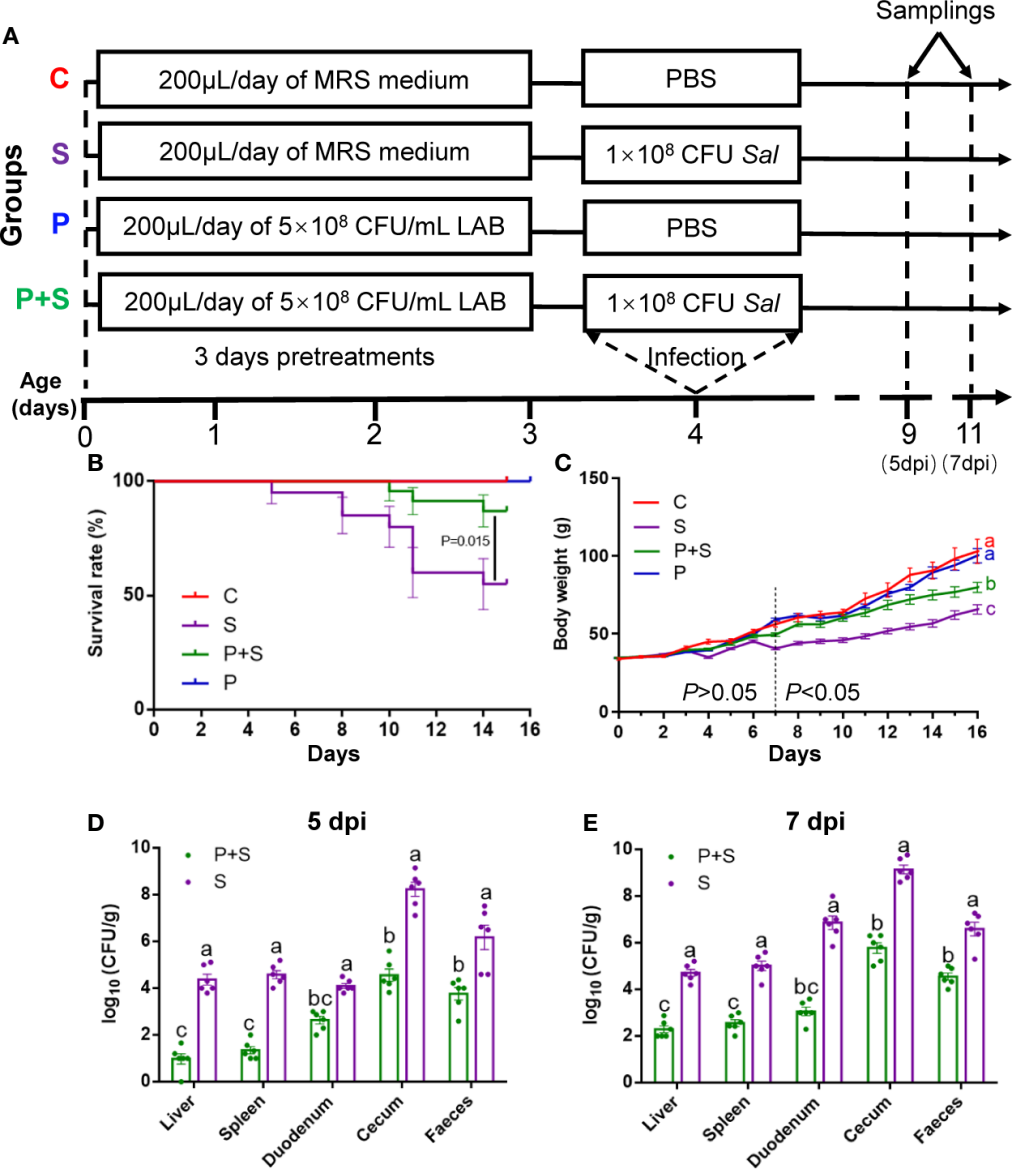
Study design and protective effect of LAB against *Salmonella* fatal infections. At the beginning of three days of age, chicks in groups P and P+S received 0.2 ml phosphate-buffered saline (PBS) containing 1 × 10^8^ CFU of the *L. rhamnosus* P118. On the fourth morning, groups S and P+S received 1 × 10^8^ CFU of the *S.* Typhimurium SL1344, *via* oral gavage. The chicks in the C group received 0.2 ml of sterile PBS **(A)**, chick survival rate **(B)**, chick growth status **(C)** and determination of bacterial load in tissues and organs infected with *Salmonella* on 5-day **(D)** and 7-day-post-infection (dpi) **(E)**. a, b, c, d: indicated the same column with different letters differ significantly (*P* < 0.05).

A total of 160 chickens, 80 were used for clinical trials and 80 were used to observe survival and growth. At the same time, the same grouping method and feeding conditions were used. One hundred and sixty 1-day-old healthy female Babcock chicks (License No.: 2021-0005) were purchased from a standardized hatchery (ChunPai hatchery, CiXi, Zhejiang, China). Four groups of chicks were randomly divided (n=40 in each group), and each chick was given its own unique number. In each group, 20 chicks are used to count the weight, growth status and survival rate, and the other 20 chicks are used to monitor clinical symptoms, and obtain tissue samples and other biomaterials used in subsequent experiments. The treatment groups were as follows: (I) the negative control (no *L. rhamnosus* P118 pretreatment and no *Salmonella* infection, designed as C); (II) the *L. rhamnosus* P118-pretreated group (10^8^ CFU *L. rhamnosus* P118, designed as P); (III) the *Salmonella*-infected group (10^8^ CFU *S.* Typhimurium SL1344, designed as S); and (IV) the *L. rhamnosus* P118-pretreated and *Salmonella*-infected group (10^8^ CFU *L. rhamnosus* P118 and 10^8^ CFU *S.* Typhimurium SL1344, designed as P+S) (**Figure 1A**). For the duration of the experiment, all chicks received free access to water and a starter feed without antibiotics. Throughout this study, all experimental protocols were approved by the Ethics Review Committee of Experimental Animal Welfare of Zhejiang University (Ethical Approval ZJU20190093) and the safety procedures were followed.

### Tissue collection and storage

At the five- and seven-day post-infection periods, six chicks from each group were selected randomly. Under respiratory anesthesia (Matrx VMR, Midmark, OH, USA), chicks were subjected to cardiac blood collection using vacuum blood collection vessels (BD Vacutainer ®, NJ, USA). Blood samples were incubated for one hour at room temperature before centrifugation at 2,000 g for 10  min to separate serum. Chickens were euthanized by cervical dislocation under anesthesia. One arm of cecum contents, duodenum, liver, spleen and fecal were collected for *Salmonella* enumeration. To prepare for the follow-up observation of intestinal ultrastructure, a segment (~1 cm) of cecum and duodenum were fixed in 2.5% glutaraldehyde solution (electron microscope fixing solution). Duodenum and jejunal mucosa were scraped using a glass slide (10 cm length), and segments were stored at −80°C for total RNA isolation. Meanwhile, a segment from another arm of the cecum was collected and separated into two parts. One part was fixed in 4% paraformaldehyde, while the other part was kept in liquid nitrogen for further research.

### Enumeration of *Salmonella*

The tissue samples (duodenum, cecum, liver, spleen, and feces) were aseptically collected from the euthanized animals at 5- and 7-days post-infection (dpi). Samples were serially diluted in sterile saline (0.9% NaCl) and aliquots were transferred to xylose lysine deoxycholate (XLD) agar (Land Bridge Co., Ltd., Beijing, China) plates supplemented with 50μg/mL nalidixic acid (BBI life science, Shanghai, China), which were incubated under aerobic conditions at 37°C for 24 h. Logarithmically transformed (log10) *Salmonella* counts were expressed in colony-forming units per gram (CFU/g). For the samples that showed no growth in the agar plate, the backup samples from the same source were partially diluted and transferred to buffered peptone water (BPW) (Land Bridge Co., Ltd., Beijing, China) incubated at 37°C for 24 hours under shaking. Then, they were plated onto brilliant green agar (Land Bridge Co., Ltd., Beijing, China) plates to detect *Salmonella*.

### Histopathology scoring

**Figure 2:**
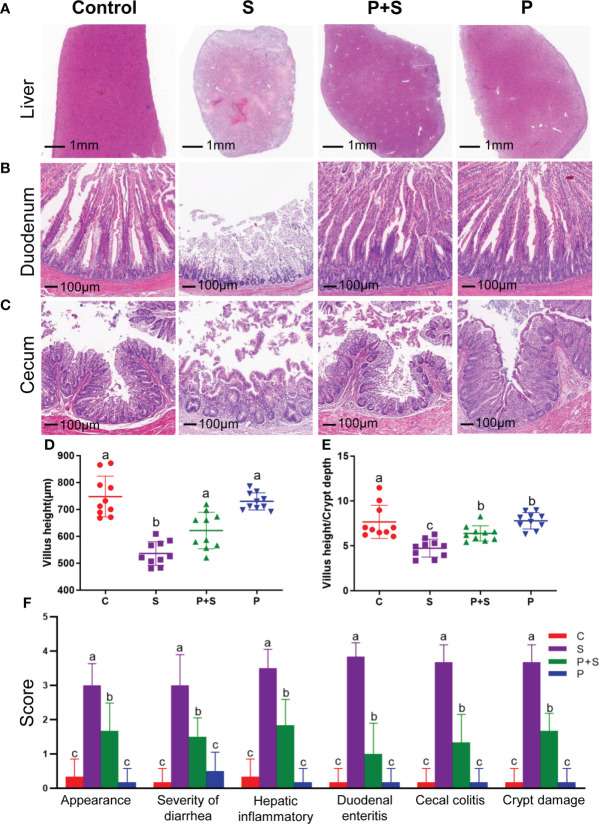
Pathological, changes of intestinal villus, clinical score and intestinal inflammation score in different groups. **(A–C)** Effects of different treatments on histopathology and **(D)** changes of villi height and **(E)** ratios of villi height to crypt depth in the duodenum, and **(F)** histological inflammation scores of each organization in newly hatched chicks. a, b, c: indicated the same item with different letters differ significantly (*P* < 0.05).

Tissue blocks from the duodenum, cecum and liver were fixed in the 4% paraformaldehyde buffer for 24 hours, rinsed with PBS repeatedly for 5 times, and then embedded in paraffin. Tissues were cut into serial sections (~5 μm thickness) and stained with hematoxylin and eosin (HE staining), the best overall quality section was used for histopathological analysis. According to Bandara et al. (39) and Memon et al. (40), two blinded investigators were scored histological inflammation and clinical appearance, including the degree of inflammation of liver and intestinal tissues, crypt damage, and the percentage of involvement of inflammatory area in each slide (**Table 1**, **Figures 2A–C**).

**Table 1:** Gross and microscopic pathology scoring criteria.

Score	Macroscopic		Microscopic
	Appearance performance	Severity of diarrhea	Inflammatory infiltration or hemorrhage
0	None	None	None
1	Listlessness	Minimal (Semi solid shape)	Minimal (<5% of section)
2	Messy feathers	Mild (Semi solid mixed with foam liquid)	Mild (5–10% of section)
3	Difficult to stand	Moderate (Liquid feces)	Moderate (11–30% of section)
4	Weak breathing	Marked (Bloody diarrhea)	Marked (>30% of section)

### Transmission electron microscope (TEM) observation

We performed samples according to the Zhejiang University Bio-ultrastructure analysis Lab’s rules for animal sample preparation. In brief, the samples underwent double fixation, infiltration, embedding, ultrathin sectioning and staining, and were observed in H-7650 TEM (Hitachi, Ltd., Tokyo, Japan). Reagents used in the preparation process as glutaraldehyde, PBS, ethanol, acetone, lead citrate and methylene blue are all produced by Sinopharm Chemical Reagent Co., Ltd., Shanghai, China. Osmic acid, Spurr resin embedding agent and uranyl acetate are produced by Structure Probe, Inc. West Chester, PA, USA.

### Immunohistochemical analysis

Histomorphological status was examined by HE-stained sections as described previously. Then the paraffin sections are dewaxed to water, and the tissue sections are placed in EDTA buffer (pH=9.0) at 95°C for antigen repair and maintained in sub boiling state for 15 minutes. It is important to avoid excessive evaporation of the buffer and to dry the slices as little as possible during this process. After natural cooling, the slices were placed in PBS (pH=7.4) and shaken for 15 min for decolorization. Then they were put into the 3% hydrogen peroxide solution and incubated at room temperature in the dark for 25 min to block endogenous peroxidase. Afterwards, they were placed in PBS (pH=7.4), shaken and washed for 15min. Further, 3% BSA was added dropwise to cover the slices evenly, and the serum was blocked at room temperature for 30 min. Then the blocking solution was removed, the diluted primary antibody (1:50) drops were added (Servicebio® Co., Ltd., Wuhan, China) to the slices, and the slice flat was placed in the wet box for incubation at 4°C overnight. The next day, the slice was placed in PBS (pH=7.4), shaken and washed for 15 min. After the sections were dried, the tissues were covered with secondary antibodies (1:500, HRP labelled) corresponding to the primary antibody and incubated at room temperature for 50 min. Next, after washing and drying the slices, the freshly prepared diaminobenzidine (DAB) color developing solution was added, the control of color development time was done under the microscope (the positive is brown-yellow), and slices were washed with distilled water to stop the color development.

Then the hematoxylin was counterstained for 3 min, washed with distilled water, and treated with hematoxylin differentiation solution for 10 s. Washing with distilled water, the hematoxylin returned to blue. Finally, to dehydrate the slice and make it transparent, the slices were put into 75% alcohol for 5 min, 85% alcohol for 5 min, 100% ethanol I for 5 min, 100% ethanol II for 5 min, n-butanol for 5 min, xylene I for 5 min. The slices were taken out from xylene, dried slightly, and sealed. After completing the above steps, the microscopic examination, image acquisition and analysis were operated. In the visual field, hematoxylin-stained nuclei are blue, and the positive expression of DAB is brownish-yellow.

Finally, the apoptotic cells and some tight junction protein aggregates were quantitatively analyzed with the aid of a light microscope (Olympus, Tokyo, Japan), which was equipped with a digital camera and an image analysis program (Image-Pro ® Plus version 6 software, Media Cybernetics, MD, USA). The data were showed as the number of stained cells per 1,000 μm2 in the villus and crypt regions.

### Serum parameters measurement

The endotoxin activity (ET) was measured according to the instruction of the kit (Xiamen Tachypleus amebocyte lysate Reagent Co., Ltd., Fujian, China), and the activity was expressed in EU/mL. The cytokine D-lactic acid in serum was determined according to the procedure of the commercial ELISA kit (Jiancheng Bioengineering Institute, Nanjing, China). The competition method was used to detect the content of D-lactic acid in the sample. The serum was added into the enzyme-labelled hole pre-coated with antibody, and the recognition antigen labelled by horseradish peroxidase (HRP) was added. When incubated at 37°C for 1 hour, they compete with solid-phase antibodies to form immune complexes. After washing with PBS, the bound HRP catalyzes TMB (tetramethylbenzidine) to turn blue, then terminates the reaction under the action of sulfuric acid to turn yellow. There is an absorption peak at the wavelength of 450 nm. The absorbance value is inversely related to the antigen concentration in the sample.

The contents of immune-related factors IL-1β and IL-18 in serum were determined according to the operation manual of the commercial ELISA Kit (Solarbio® Sci & Tech Co., Ltd., Beijing, China). In short, they use the enzyme-linked immunosorbent assay based on the double antibody sandwich method to coat the monoclonal antibodies against chicken IL-1β and IL-18 on the enzyme label plate and add the standard and sample, respectively. The IL-1βand IL-18 in the sample will fully bind to the coated antibody. After washing with PBS, add a biotinylated secondary antibody, and the secondary antibody will specifically bind to IL-1β and IL-18. After washing with PBS, TMB is added, and the combined HRP catalyzes TMB to turn blue. After adding sulfuric acid termination solution, it turns yellow. The absorbance of the reaction pore sample was measured at 450 nm. The contents of IL-1β and IL-18 in the sample were positively correlated with the OD value. The contents of IL-1β and IL-18 in serum can be obtained by drawing a standard curve and four-parameter fitting calculation.

### RNA isolation and quantitative real-time PCR

Total RNA of cecum samples was extracted using RNA Easy Fast animal tissue total RNA Extraction Kit (TIANGEN® Biotech, Beijing, China) according to the manufacturer’s protocol. The concentration and purity of total RNA were quantified with a NANODrop® ND-1000 UV-VIS spectrophotometer (Thermo Scientific, Wilmington, DE, USA) and agarose gel electrophoresis. The quantitative real-time PCR assay was performed with the Stratagene Mx3005P (with Mxpro 4.10 software) detection system (Agilent Technologies, Santa Clara, CA, USA) according to optimized PCR protocols using the SYBR qPCR Master Mix kit (Vazyme Biotechnology Co., Ltd., Nanjing, China). The primer pairs for amplifying genes encoding tight junction protein-related genes Claudin-1 and ZO-1, apoptosis-related gene Bax, and inflammasome-related gene IL-1β, and IL-18 are presented in **Table 2**. GAPDH was used as an internal reference. The qPCR conditions were an initial denaturation step at 95°C for 30 s, 40 cycles at 95°C for 5 s, and annealing and extension temperature at 55-60°C (**Table 2**) for 35 s. Each biological sample was run in triplicate. The method of 2−ΔΔCt (46) was used to calculate relative gene expression levels between different samples.

**Table 2:** Primers used to analyze gene expression by quantitative RT-PCR.

Gene	Primer sequence (5′–3′)	Size (bp)	Annealing temperature(°C)	Reference	GenBank No.
Claudin-1	F: CTGATTGCTTCCAACCAG	140	59	(41)	NM_001013611
	R: CAGGTCAAACAGAGGTACAAG				
ZO-1	F: CTTCAGGTGTTTCTCTTCCTCCTC	131	59	(41)	XM_413773
	R: CTGTGGTTTCATGGCTGGATC				
Bax	F: GTGATGGCATGGGACATAGCTC	90	59	(42)	XM_015290060
	R: TGGCGTAGACCTTGCGGATAA				
IL-1β	F: CTGGGCATCAAGGGCTACAA	131	60	(43)	NM_204524
	R: CGGTAGAAGATGAAGCGGGT				
IL-18	F: TAACAGATCAGGAGGTGAAATCT	299	60	(44)	/
	R: AAGGCCAAGAACATTCCTTGTT				
GAPDH	F: TGGAGAAACCAGCCAAGTAT	145	55	(45)	NM_204305.1
	R: GCATCAAAGGTGGAGGAAT				

### Intestinal flora diversity

#### DNA extraction

Five samples of cecum contents per group were randomly selected to proceed to full-length 16S rRNA sequencing. Bacterial DNA from the ileal and cecal digest was extracted using an E.Z.N.A.® Bacterial DNA Kit (Omega Bio-tek, Inc. Norcross, GA, USA). The concentration and purity of DNA were measured using the NANODrop® ND-1000 spectrophotometer (Thermo Scientific, Wilmington, DE, USA), and the DNA quality was determined by agarose gel electrophoresis.

#### Full-length 16S sequencing analysis

The sequencing library was obtained according to the 16S Amplification SMRTbell® Library Preparation workflow. The library was sequenced on the PacBio Sequel II platform (Magigene Biotechnology Co., Ltd. Guangzhou, China). PacBio data was processed by SMRT link (version 6.0) software, including data splitting, sequence error correction, and sequence format conversion. Finally, clean data was obtained. The sequences were clustered into operational taxonomic units (OTUs) based on a 97% similarity threshold with the UPARSE-ver10 (http://www.drive5.com/usearch/). During the clustering, UPARSE could remove the chimera sequence and singleton OTU at the same time. The taxonomic information for each representative sequence was annotated by mapping the silva-ver132 (https://www.arb-silva.de/) database. In order to study the phylogenetic relationship of different OTUs, the KRONA software (http://sourceforge.net/projects/krona/) was used to visualize the results of individual sample annotations. GraPhlAn (http://huttenhower.sph.harvard.edu/graphlan) was used to get a single sample OTU annotation circle graph to know the species composition and abundance information in the sample.

Alpha diversity was used to analyze the complexity of species diversity for each sample through 3 indices, including ace and Chao1. All these indices were calculated by QIIME V1.9.1. Three non-parametric analyses (analysis of similarity, ANOSIM); non-parametric multivariate analysis of variance, Adonis; a multiresponse permutation procedure, MRPP, were performed by R software based on the OTU table to display the extent of differences between groups. LDA Effect Size (LEfSe) analysis was used to find the biomarker of each group.

### Data analysis

The data from the experiment were subjected to ANOVA after determining variance homogeneity by using the SPSS 16.0 software (IBM, New York, USA). The figures for data visualization were performed using GraphPad Prism 9.3 (GraphPad Software Inc., San Diego, CA). The analysis of other data was performed using Student’s t-test. The difference was considered to be statistically significant as P < 0.05, and the data were expressed as the means ± SEM.

## Results

### Survival rate and growth status of chicks

To explore the effect of *L. rhamnosus* P118 on reducing *Salmonella* infection in chicks, according to our experimental plan, we used 160 female chicks, of which 80 were used to observe survival and growth, with 20 in each group. Another 80 were used for clinical trials (**Figure 1A**). They were equally divided into four groups and received the same environment and care as noted. It can be seen that on the 15th day, the mortality of chicks in group S was close to 50.00%, higher than that observed in group P+S pretreated with probiotic P118 (15.00%) (P <0.05), while no death was observed in groups C and P (**Figure 1B**). As shown in **Figure 1C**, there was no statistical difference in daily gain between groups C and P (P >0.05). On the other hand, chicks infected with *Salmonella* grow slowly, especially after six days. There was no significant difference in body weight between the treatment groups at or before the age of 6 days. However, from the age of 7 days to the end of the experiment, the difference between the groups was significant (P <0.05) and gradually increased. Due to the early intervention of *L. rhamnosus* P118, even after the challenge, the adverse situation of *Salmonella* infection in the P+S group was reversed.

### Colonization of tissues and feces by *S.* Typhimurium

To determine the effect of the oral *Salmonella* challenge, the loads of *S.* Typhimurium in the liver, spleen, duodenum, cecum and feces were determined. *S.* Typhimurium was not detected in the tissue samples from the C and P groups. In comparison with the S group, the number of *S.* Typhimurium colonized in the tissues and feces was significantly lower in the P+S group (P < 0.05) (**Figures 1D, E**). Moreover, we found that when compared with five dpi, the clinical symptoms and organ bacterial loads of chicks on day seven dpi were significantly (P <0.05) different between the groups (**Figures 1D, E**). Therefore, the pathological sections, serum biochemical indexes, determination of immune factors, immunohistochemical analysis, full-length 16S rRNA sequencing and other tests mentioned later are all based on the chicks’ samples on day seven dpi.

### Histopathology and intestinal microstructure

In order to determine if liver and intestinal integrity were affected by *S.* Typhimurium infection, the morphology of samples was monitored. Only the S group showed large number of heterophils, indicating a mild inflammatory response at seven dpi. Hematoxylin-eosin-stained pathological sections showed that chicks in group S presented a disordered structure of hepatic cord, unclear structure of hepatic lobules, narrowing or even disappearance of hepatic sinuses, loose spacing of hepatocytes and a large number of pink eosinophilic particles (**Figure 2A**). *S.* Typhimurium caused severe damage to the villi morphological structure of the duodenum and cecum (**Figure 2B, C**). The integrity of the intestinal inner wall of the duodenum in group S was damaged, eosinophils were found in the inner wall cells, duodenal mucous gland cells disappeared, the morphology of intestinal villi was seriously degraded, some crypt structures were unclear, and the relative depth of the surviving crypts increased (**Figure 2B**). In group S, the cecal villi were also significantly shorter, some epithelium on the mucosal surface was missing, inflammatory cells in lamina propria were increased, and neutrophil infiltration was prominent. Still, the overall structure was visible (**Figure 2C**). It significantly reduced the height of the duodenal villi and increased the depth of recess (**Figures 2D, E**, **Table S2**). Moreover, *S.* Typhimurium strongly increased (P < 0.05) the appearance score, inflammation score, diarrhea score and crypt damage score. *L. rhamnosus* P118 pretreatment (P + S) significantly (P < 0.05) reversed the trend and decreased all the group’s scores compared with the S group. (**Figure 2F**, **Table S1**).

**Figure 3:**
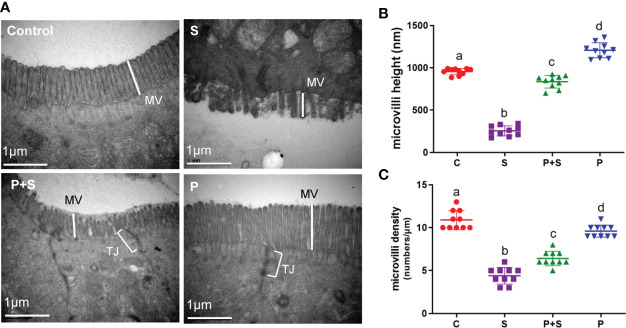
The structural damage in intestinal microvilli and microvilli length and density changes. **(A)** The effects of different treatments on microvilli (MV) structure and tight junction (TJ) protein in the chicken intestinal tract were observed under a transmission electron microscope (TEM). **(B, C)** The length and density of microvilli were measured. a, b, c, d: indicated the same item with different letters differ significantly (*P* < 0.05).

Based on this result, we attempted to visualize microvilli ultrastructure in high-resolution transmission electron microscopy (TEM) to examine the effects of *L. rhamnosus* P118 stimulation on intestinal microvillus development (**Figure 3A**). The results showed that the microvilli of the chicks treated with *L. rhamnosus* P118 had a significantly 1.3 times longer length (1208.4 ± 28.02 nm) (**Table S2**) and more perpendicular to the top surface of intestinal cells compared with the C group (**Figures 3A, B**). The density of microvilli was higher than P+S (P< 0.05) (**Figure 3C**, **Table S2**).

### Detection of proteins and genes related to intestinal barrier function

**Figure 4:**
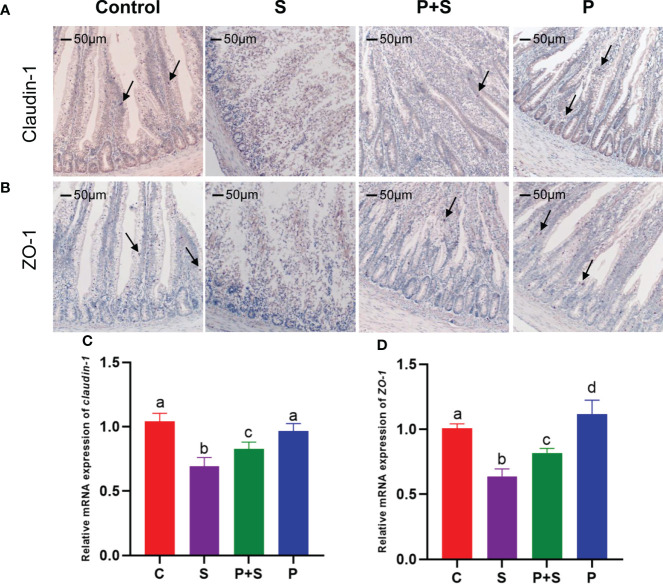
Immunohistochemical staining sections of tight junction and the relative genes expression. Immunohistochemical staining was used to identify the presence of tight junction proteins **(A)** claudin-1 and **(B)** ZO-1 in intestinal tissues, and RT-qPCR was used to detect **(C, D)** the relative mRNA expression of tight junction protein genes. The arrow in the figure refers to a part of the stained tight junction protein. a, b, c, d: indicated the same item with different letters differ significantly (*P* < 0.05).

To determine that the morphological changes in the chicken intestinal tract are indeed affected by barrier function-related proteins, claudin-1 and ZO-1 were used for immunohistochemical staining of the duodenum. **Figures 4A, B** shown that these two proteins will be dyed brown yellow. The arrow in the figure refers to a part of the stained tight junction protein.

To investigate why *S.* Typhimurium and *L. rhamnosus* P118 changed the intestinal permeability, and the expression of TJ genes claudin-1 and ZO-1 were measured by qPCR. As shown in **Figure 4C**, there was a significant difference in claudin-1 and ZO-1 at the mRNA level among all four groups (P < 0.05). In comparison with the C group, *Salmonella* infection (group S) significantly (P <0.05) decreased the mRNA levels of ZO-1 and claudin-1(**Figures 4C, D**). The down-regulation of ZO-1 and claudin-1 due to *Salmonella* infection was eliminated in the P+S group. Moreover, in the group P, the ZO-1 expression was significantly (P <0.05) higher than that in group C (**Figure 4D**).

### Detection of blood biochemical indexes of chicks

**Figure 5:**
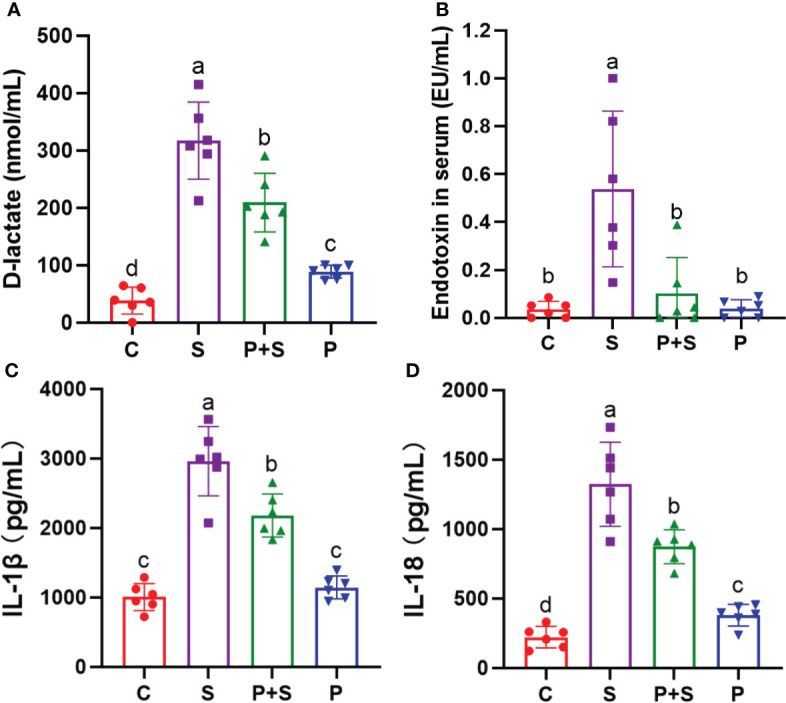
Determination of intestinal barrier function factors and immune function factors in chicken serum. The contents of **(A)** D-lactic acid and **(B)** endotoxin in chicken blood were determined by ELISA, which can reflect the state of intestinal injury. The contents of **(C)** IL-1β and **(D)** IL-18 in chicken blood were measured by ELISA to evaluate the degree of inflammatory response. a, b, c, d: indicated the same item with different letters differ significantly (*P* < 0.05).

To assess the intestinal permeability among different treated groups, levels of mediators D-lactic acid and endotoxin (ET) in serum samples were determined. As shown in **Figures 5A, B** *S.* Typhimurium infection significantly (P <0.05) increased intestinal permeability compared with the C group. However, the D-lactic acid and ET in the serum of the chicks in the P+S group were significantly lower than those in the S group (P < 0.05). There was no significant difference between the P group and the P+S group in endotoxin (P > 0.05) (**Table S3**).

### Detection of immunological factors from chick blood

To elucidate changes in the inflammatory responses induced by *Salmonella* infection, ELISA was used to measure the levels of cytokines IL-1β and IL-18 in serum samples (**Figures 5C, D**). Compared with the C group, *Salmonella* infection significantly increased the levels of cytokines in serum (P < 0.05). However, after probiotic pretreatment, the levels of IL-1β and IL-18 were significantly decreased in the P+S group compared with the S group (P < 0.05) (**Figures 5C, D**, **Table S4**).

### Expression of apoptosis-related proteins

**Figure 6:**
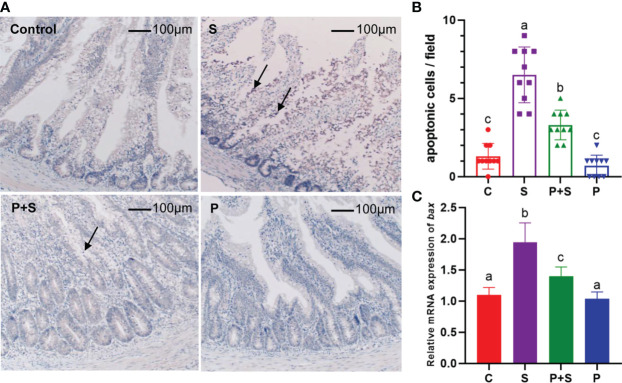
Immunohistochemical staining sections of apoptosis-related proteins and the relative gene expression. **(A)** Immunohistochemical staining was used to identify the existence of apoptosis in intestinal tissue, the arrow points to a brown-stained apoptotic protein aggregation region. **(B)** The average number of apoptotic cells was counted in each field. **(C)** RT-qPCR was used to detect the relative mRNA expression of gene *Bax*. a, b, c: indicated the significant differences among groups (*P* < 0.05).

Apoptosis also affects intestinal barrier function. Apoptotic cells were found in the S group, and P+S group, as shown in **Figure 6A**, the cytoplasm of cells dyed brown-yellow is apoptotic cells containing Bax gene expression products. The more cells stained per unit area, the higher the frequency of apoptotic events. After *Salmonella* infection, the number of apoptotic cells in group S was significantly (P < 0.05) higher than that in the control group (**Figure 6B**).

To investigate whether *Salmonella* infection caused apoptosis in chicks, immunohistochemical staining and qPCR were also used to detect the levels of apoptosis-related proteins and mRNA. Compared with group S, the process of apoptosis was significantly (P <0.05) reduced after probiotic pretreatment. The change in the trend of the mRNA expression level of Bax is mutually confirmed with the above results, it was highly expressed in group S and significantly (P < 0.05) higher than in other treatment groups. Similarly, *L. rhamnosus* P118 pretreatment improved this trend (**Figure 6C**).

### Changes in chicken intestinal flora

**Figure 7:**
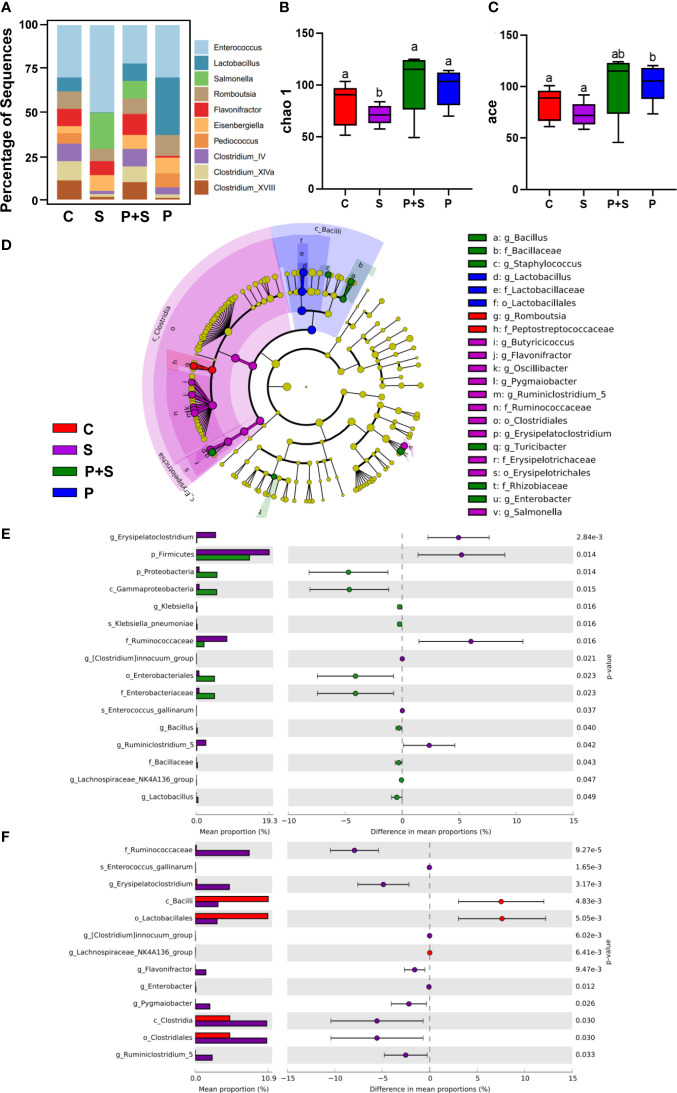
Full-length 16S rRNA sequence analysis of cecal contents in chicks and the microflora data alpha- and beta-diversity analysis. **(A)** Comparison of the top ten distribution of bacterial communities in different treatment groups at the genus level. **(B, C)** Analysis of alpha diversity index of chicken intestinal flora under different treatment conditions. **(D)** Five-level cladogram of microbial communities in different treatment groups (threshold score > 2). The circle radiating from inside to outside represents the taxonomic level from phylum to genus (or species). Each small circle at different classification levels represents a classification at that level, and the diameter of the small circle is directly proportional to the relative abundance. The species with no significant difference are marked as yellow. The biomarker of the different species is the same as that of legend. **(E)** The top 16 differentially abundant bacteria at genus level in S vs P+S groups. If the former was not obtained, the classification at the genus level or the family/order level. Log scale was used in the abscissa. **(F)** The top 13 differentially abundant bacteria in S vs C groups at the genus level. If the former was not obtained, the classification at the genus level or the family/order level. Log scale was used in the abscissa. s_= species; g_ = genus; f_ = family; o_ = order; c_ = class; p_ = phylum. a, b, c, d: Means in a same item with different letters differ significantly (*P* < 0.05).

In terms of the genus of full-length 16s rRNA sequencing (**Figure 7A**), we highlight the marked increase in Lactobacillus spp. in the feces of chicks pretreated with *L. rhamnosus* P118 (32.15%) in group P compared with S (1.10%), P+S (10.10%), and C (6.79%) groups. Variations in the abundance of *Salmonella* across the groups were also observed: group P+S showed significantly decreased abundance (9.88%) compared with group S (19.85%), while the *Salmonella* spp. was not detected in group C and group P. Interestingly, in the *Salmonella* infection group, the proportion of *Salmonella* is only the second, and the dominant genus is still *Enterococcus*, and the sum of the two genera reaches 70.27% in top ten genera, which dramatically reduces the living space of other genera. Relatively, the proportion of various genera in the P+S group is balanced.

The corresponding alpha diversity analysis is shown in **Figures 7B, C**. Using the Chao 1 and ace indexes analysis, the abundance of microbes in the guts of chicks was further validated. The species diversity in chicks after *Salmonella* challenge (S group) was significantly (P < 0.05) decreased, while in the P+S group, it was increased obviously (P < 0.05) compared with the group C (ace indexes). Investigation of species and ace indexes showed that pretreatment with *L. rhamnosus* P118 significantly increased species richness and stabilized the proportion of balanced gut microbiota compared with controls (**Figure 7A, C**).

LEfSe analysis is shown in **Figure 7D**, which presents OTU at different taxonomic levels that are significantly different between group S and the other groups (**Figure S1**) (P < 0.05). On day seven dpi, when compared with the P+S, the S group showed an increase in the relative abundance of Erysipelatoclostridium and the cecum contents (**Figure 7E**, P<0.05) and a decrease in *Lactobacillus* spp. abundance in the cecum. At the same time, compared with the C group, at the genus level, it is the same as S vs P+S, but the relative abundance of *Erysipelatoclostridium* is still significant different (**Figure 7F**).

## Discussion

The intestinal mucosal barrier is considered the frontline of defense against pathogen infection, regardless of its essential role in nutrient absorption. The beneficial properties of intestinal microbiota enhance the functions of the intestinal barrier. In fact, some previous studies have approved the role of gut microbiota, especially probiotics, in maintaining the stability and vital function of the intestinal barrier (47). However, newly hatched chicks have immature immune systems (48) and under-developed gut microbiota indicated by low diversities and densities (49, 50). These weaknesses make the newly hatched chicks more susceptible to infections, especially those caused by *Salmonella*, where this bacterium damages the intestinal mucosal barrier and then induces severe intestinal inflammations and diarrhea (26, 28, 51). In this regard, different studies have reported the implication of *Salmonella* in the high mortality rate of chicks (2, 3). Prophylactic and therapeutic strategies based on antibiotic treatment are limited due to their adverse effect on the increased spread of antimicrobial resistance. Hence, the recourse to sustainable and friendly alternatives is a major priority. In this case, probiotics have proved efficacy, safety, and sustainability in different fields (52, 53).

The administration of *L. rhamnosus* reduced the mortality rate of chicks from 50.00% to 15.00% and ameliorated the body weight of *Salmonella*-infected chicks. These findings are consistent with previously reported studies, in which different probiotics have demonstrated the ability to reduce the mortality rate and to improve body weight gain of chicks infected with *Salmonella* (54–56). Additionally, our findings showed the effectiveness of *L. rhamnosus* in reducing the number of *Salmonella* colonized Liver, spleen, duodenum, cecum, and feces. As known, during the infectious pathway, *Salmonella* damages the intestinal barrier, penetrates epithelial cells, and then translocated into vital organs via bloodstream circulation, leading to systemic infection with diarrhea and the continuous shedding of bacteria in feces. In fact, reducing damage to the intestinal barrier could be a key solution in stopping the *Salmonella*-infectious pathway and then reducing the number of bacteria in organs.

Intestinal morphology, especially the length of villi and the crypt depth, is a vital index affecting the chicken intestinal tract’s health and growth performance. Histopathological and intestinal microstructure observation showed that the administration of *L. rhamnosus* P118 mitigated the severe damage caused by *S.* Typhimurium infection in the villi morphological structure of the duodenum and cecum, in addition to the adverse effects such as height reduction of duodenal villi and the increase in the depth of the recess. Chicks treated with *L. rhamnosus* P118 had higher microvilli length and density than non-treated chicks (S group). Hence, maintaining the necessary form of villi and the crypt depth may promote the chick’s growth and body health. Similarly, a study performed by Ye et al. showed that the administration of probiotic supplements had significantly increased the villus heights and the ratio of villus height to crypt depth of chicks and then increased performances in body weight and average daily gain (57). Another study by Nii et al. showed that oral administration of *Limosilactobacillus* (formerly *Lactobacillus*) *reuteri* increased ileal villus height and crypt depth of broiler chicks (58). Moreover, Wang et al. showed that prophylactic feeding of *Lacticaseibacillus casei* DBN023 to *S.* pullorum infected chicks significantly increased their jejunum villar height, villar height-to-crypt-depth ratio, and reduced intestinal-crypt depth (59).

However, microvilli cannot maintain the morphology without tight junction proteins. Tight junction proteins can form continuous intercellular contact between the intestinal epithelium and are essential to intestinal barrier function. In this study, we showed that the expression of tight junction genes, including claudin-1 and ZO-1, was significantly higher in the treated group (P+S) than in the *Salmonella*-infected group (S). A study by Qin et al. found that *Lactiplantibacillus plantarum* alleviated *Salmonella*-induced dextran permeability and decreased ZO-1 proteins in Caco-2 cells (60). Bhat et al., and Mohd et al., found that *L. rhamnosus* and *L. fermentum* significantly improved the *E. coli*-disturbed tight junction proteins (Occludin, ZO-1, claudin-1) in Caco-2 cells (47, 61). Additionally, Deng et al. showed that *L. casei* expanded tight junction protein levels, including ZO-1 and Claudin-1 (62). Moreover, Nii et al. found that oral administration of *L. reuteri* increased the expression of Claudin-1, Claudin-5, ZO-2, and JAM2 in broiler chicks infected with *S.* Typhimurium (63). Hence, our findings evidenced that the administration of *L. rhamnosus* P118 protected the intestinal epithelial barrier from *Salmonella* Typhimurium infection by regulating the expression of tight junction relation genes. However, the mechanisms behind this regulation by *L. rhamnosus* are still not well understood. Furthermore, pretreatment of *Salmonella*-infected chicks with *L. rhamnosus* P118 reduced the proportion of *Salmonella* in the gut, which could be another potential mechanism by which probiotics protect the intestinal barrier integrity.

The infection of chicks by *Salmonella* induces inflammatory responses. Cytokines play an irreplaceable role in immune response and inflammation in chicks to *Salmonella* infection (64). In this study, we showed a high producing level of pro-inflammatory IL-1β and T helper (Th1) cytokine IL-18 in chicks infected with *Salmonella*, while this level was significantly reduced after the administration of *L. rhamnosus* P118. The reduction of IL-1β and IL-18 levels may be explained by the mitigation of intestinal damages caused by *Salmonella* after the administration of *L. rhamnosus* P118 and the reduction of inflammatory response. The results suggest a cytokine-mediated immune response mechanism against *Salmonella* infection in the intestinal epithelium of chicks. Similarly, a study conducted by Chen et al. showed that the administration of a mixture of lactic acid bacteria reduced the level of IL-1β, IL-6, and IFN-γ in *Salmonella* infected-chicks (65). These data agree with previous findings where the IL-1β and IL-18 genes were up-regulated in the spleen and cecum of new hatch chicks after oral inoculation with *Salmonella* (66). Furthermore, it has been previously reported that the mRNA expression of IL-1β, IFN-γ and IL-18 were significantly up-regulated in the cecal tonsil of *Salmonella* challenged hens (67). The data from the current study suggest that the intestinal epithelium was capable of initiating a cellular immune response and a Th1-cytokines reaction to *Salmonella* Typhimurium through activation of specific cytokine genes.

The diversity and balance of gut microbiota are essential for maintaining the beneficial functions of the intestinal mucosal barrier and the host’s health, which may improve the host’s immune system and then the host’s resistance to infection (68). At the same time, the microbial community’s disruptions translate into host susceptibility alterations, especially in enteric infections (69). It has been pointed out that the normal gut microbial of chicks are rich in probiotic bacteria, including *Lacticaseibacillus* and *Bifidobacterium*, which may be contribute to intestinal homeostasis, preventing the invasion of pathogenic bacteria in a proactive way (70). However, the high abundance of Bacteroides disrupted the gut microbiota balance, which further stimulates the inflammatory response and causes secondary infection with other pathogens (71). In this study, we showed that the administration of *L. rhamnosus* P118 decreased the proportion of *Salmonella* and balanced the diversity of gut microbiota disrupted by the *S.* Typhimurium infection. Indeed, maintaining the microbiota balance may improve the host immune system and then reduce the *Salmonella* infection. Similarly, a recent study by Khan and Chousalkar (72) showed that probiotic-based *Bacillus* improved the diversity and abundance of gut microbiota displayed by the *Salmonella* challenge in chickens. Notably, the mechanisms by which probiotics maintain the gut microbiota balance and reduce the load of pathogens are diverse, heterogeneous and may be strain-specific (73), which may be linked to the production of organic acids, activation of the host immune system, and the production of antimicrobial agents (74). Moreover, the competition for vital nutrients can be one of these mechanisms. Deriu et al. (75) demonstrated that the competition on the iron acquisition by the probiotic *E. coli* strain Nissle 1917 had reduced *S.* Typhimurium colonization in mouse models.

Nevertheless, some remaining points could be further studied. Since we have only quantified certain inflammation-related factors and investigated a reduction in intestinal cell apoptosis, the exact mechanisms by which *L. rhamnosus* P118 exerts its anti-inflammatory effects is an ongoing question. Our findings provide evidence that *L. rhamnosus* P118 significantly reduce intestinal cell apoptosis, but the potential mechanism also remains to be further study. Together, our data showed that the introduction of *L. rhamnosus* P118 could aid in the maintenance of the intestinal mucosal barrier and modulate the microbiota, the likely metabolites from *L. rhamnosus* P118 could be an exciting point in future studies.

## Conclusions

In this study, the effects of *S.* Typhimurium infection on the epithelial integrity of new hatch chicks were evaluated by macroscopic pathological section analysis, determination of intestinal structure under the electron microscope, determination of immune-related factors, D-lactic acid and endotoxin in serum. In addition, this study also used full-length 16S rRNA sequencing to evaluate the intestinal microbial diversity and stability of chicks infected with *S.* Typhimurium. In summary, *Salmonella* disrupted the intestinal epithelial barrier in newly hatched chicks by bacterial translocation, stimulating the inflammatory response and reducing intestinal cell apoptosis and the richness of intestinal flora. After pretreatment with *Lacticaseibacillus rhamnosus* P118, newly hatched chicks can be protected from the destruction of intestinal epithelial barrier induced by *Salmonella* by enhancing the immune wall, stabilizing the expression of tight junction, reducing intestinal cell apoptosis, reducing *Salmonella* colonization and maintaining the diversity and stability of intestinal flora.

## Data availability statement

The original contributions presented in the study are publicly available. This data can be found in NCBI under accession number PRJNA848970, https://www.ncbi.nlm.nih.gov/bioproject/PRJNA848970/.

## Ethics statement

The animal study was reviewed and approved by ethics review committee of Experimental Animal Welfare of Zhejiang University (Ethical Approval ZJU20190093).

## Author contributions

Conceptualization, MY. Performed the experiments, XP, YS, and XZ. Data analysis, XP and ME. Writing—original draft preparation, XP. Writing—review and editing, XP, AE-D, RN, and MY. Supervision, MY. Funding acquisition, MY. All authors have read and agreed to the published version of the manuscript.

## Funding

This work was supported by the National Program on Key Research Project of China (2019YFE0103900) as well as the European Union’s Horizon 2020 Research and Innovation Programme under Grant Agreement No. 861917 – SAFFI, Zhejiang Provincial Natural Science Foundation of China (LR19C180001) and Zhejiang Provincial Key R&D Program of China (2022C02024, 2021C02008, 2020C02032).

## Acknowledgments

The authors thank Bio-ultrastructure analysis Lab. of Analysis Center of Agrobiology and Environmental Sciences, Zhejiang University.

## Conflict of interest

The authors declare that the research was conducted in the absence of any commercial or financial relationships that could be construed as a potential conflict of interest.

## Publisher’s note

All claims expressed in this article are solely those of the authors and do not necessarily represent those of their affiliated organizations, or those of the publisher, the editors and the reviewers. Any product that may be evaluated in this article, or claim that may be made by its manufacturer, is not guaranteed or endorsed by the publisher.

## Supplementary material

Supplementary Figure 1 Linear discriminant analysis (LDA) was used to classify chicken intestinal flora under different treatment conditions and evaluate the influence of species with significant differences.
