# Supplementary figures and images for: Lacticaseibacillus rhamnosus alleviates intestinal inflammation and promotes microbiota-mediated protection against Salmonella fatal infections
Source: Front Immunol. 2022 Aug 11;13:973224. doi: 10.3389/fimmu.2022.973224 (PMC9411107; doi:10.3389/fimmu.2022.973224)

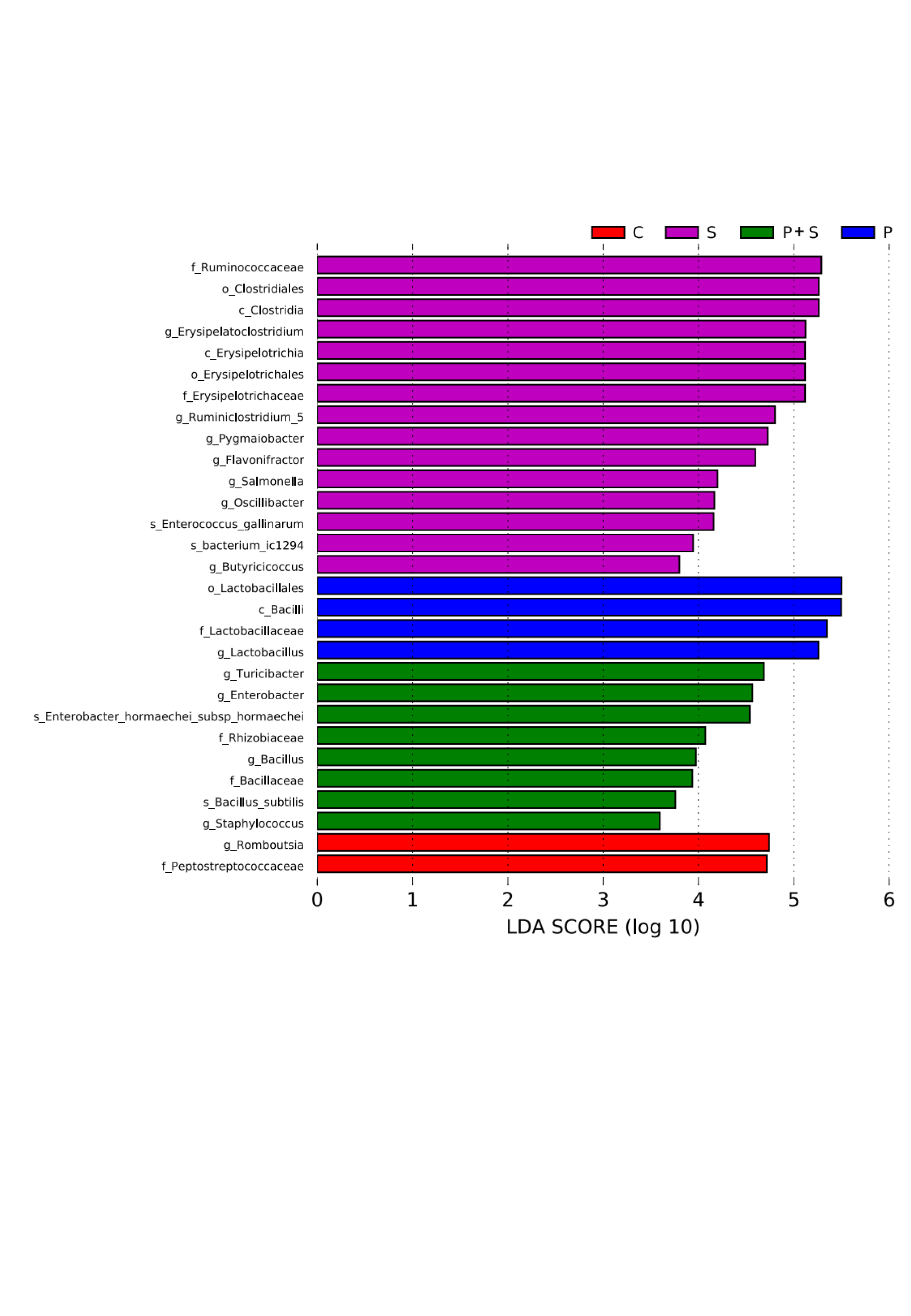

Supplement: Supplementary Figure 1 — Linear discriminant analysis (LDA) was used to classify chicken intestinal flora under different treatment conditions and evaluate the influence of species with significant differences. [file Image_1.tif]
