# Supplementary material for: Lacticaseibacillus rhamnosus alleviates intestinal inflammation and promotes microbiota-mediated protection against Salmonella fatal infections
Source: Front Immunol. 2022 Aug 11;13:973224. doi: 10.3389/fimmu.2022.973224 (PMC9411107; doi:10.3389/fimmu.2022.973224)
Supplement: Supplementary file 2 [file DataSheet_1.docx]

**Table S1.** Histological inflammation scores of each organization in newly hatched chicks.

| **Items** | **C** | **S** | **P+S** | **P** |
| --- | --- | --- | --- | --- |
| Appearance | 0.33±0.21^c^ | 3.00±0.26^a^ | 1.67±0.33^b^ | 0.17±0.17^c^ |
| Severity of diarrhea | 0.17±0.17^c^ | 3.00±0.37 ^a^ | 1.50±0.22^b^ | 0.50±0.22^c^ |
| Hepatic inflammatory | 0.33±0.21^c^ | 3.50±0.22 ^a^ | 1.80±0.31^b^ | 0.17±0.17^c^ |
| Duodenal enteritis | 0.17±0.17^c^ | 3.83±0.17 ^a^ | 1.00±0.37^b^ | 0.17±0.17^c^ |
| Cecal colitis | 0.17±0.17^c^ | 3.67±0.21 ^a^ | 1.33±0.33^b^ | 0.17±0.17^c^ |
| Crypt damage | 0.17±0.17^c^ | 3.67±0.21 ^a^ | 1.67±0.21^b^ | 0.17±0.17^c^ |

Values with different letters (a, b, c) differed significantly within an item (P < 0.05).

The data were expressed as mean ± SEM.

**Table S2.** Determination of duodenal villus length (μm), crypt depth (μm), microvilli length(nm) and villus height / crypt depth ratio in chicks.

| **Items** | **C** | **S** | **P+S** | **P** |
| --- | --- | --- | --- | --- |
| Villus height | 747.90±23.93^a^ | 536.10±13.97^b^ | 621.60±21.49^a^ | 758.00±12.23^a^ |
| Crypt depth | 100.70±5.36^a^ | 117.40±7.89^b^ | 98.30±3.76 ^a^ | 94.70±3.15 ^a^ |
| Villus height / Crypt depth | 7.67±0.59 ^a^ | 4.74±0.31 ^c^ | 6.38±0.27 ^b^ | 8.09±0.32^a^ |
| Microvilli length | 955.7±12.00^b^ | 255.2±18.81^d^ | 835.9±23.65^c^ | 1208.4±28.02^a^ |

Values with different letters (a, b, c) differed significantly within an item (P < 0.05).

The data were expressed as mean ± SEM.

**Table S3.** Contents of D-lactic acid (nmol/mL) and endotoxin (EU/mL) in serum of chicks under different treatment methods.

| **Items** | **C** | **S** | **P+S** | **P** |
| --- | --- | --- | --- | --- |
| D-lactic acid | 38.32+9.54^d^ | 317.17±27.47^a^ | 209.33±20.85 ^b^ | 88.77±4.75^c^ |
| Endotoxin | 0.04±0.01^b^ | 0.54±0.13^a^ | 0.10±0.06 ^b^ | 0.04±0.01 ^b^ |

Values with different letters (a, b, c, d) differed significantly within an item (P < 0.05).

The data were expressed as mean ± SEM.

**Table S4.** Contents of IL-1β (pg/mL) and IL-18 (pg/mL) in serum of chicks under different treatment methods.

| **Items** | **C** | **S** | **P+S** | **P** |
| --- | --- | --- | --- | --- |
| IL-1β | 1003.67±79.64^d^ | 2960.00±203.73^a^ | 2178.17±126.63^b^ | 1142.83±67.35^c^ |
| IL-18 | 221.33±31.51 ^d^ | 1321.67±123.18^a^ | 872.17±49.83 ^b^ | 379.33±31.65 ^c^ |

Values with different letters (a, b, c, d) differed significantly within an item (P < 0.05).

The data were expressed as mean ± SEM.
